# Use of an iPad App (Aid for Decision-making in Occupational Choice) for Collaborative Goal Setting in Interprofessional Rehabilitation: Qualitative Descriptive Study

**DOI:** 10.2196/33027

**Published:** 2021-11-18

**Authors:** Carla Strubbia, William MM Levack, Rebecca Grainger, Kayoko Takahashi, Kounosuke Tomori

**Affiliations:** 1 Department of Medicine University of Otago Wellington New Zealand; 2 Department of Occupational Therapy School of Allied Health Science Kitasato University Tokyo Japan; 3 Department of Occupational Therapy Tokyo University of Technology Tokyo Japan

**Keywords:** rehabilitation, goals, digital technology, mobile health, mobile phone

## Abstract

**Background:**

Goal setting is a key part of the rehabilitation process. The use of technology and electronic tools such as smartphone apps and websites has been suggested as a way of improving the engagement of users in meaningful goal setting and facilitating shared decision-making between patients and health professionals.

**Objective:**

This study aims to describe experiences of health professionals and patients in the use of the English language version of the iPad app Aid for Decision-making in Occupational Choice (ADOC) to facilitate collaborative goal setting in rehabilitation.

**Methods:**

We recruited participants from 3 acute and postacute care rehabilitation wards in both public and private organizations in New Zealand. Participants were registered allied health professionals, including physiotherapists, occupational therapists, and speech-language therapists, who engage in goal setting as part of their normal work, and their adult patients. We collected data via semistructured interviews to gather information about the experiences of the participants in the use of ADOC for goal setting. Data were analyzed with thematic analysis.

**Results:**

A total of 8 health professionals and 8 patients participated in the study. Six main themes emerged from the data: changing patients’ perspective on what is possible, changing health professionals’ perspective on what is important, facilitating shared decision-making, lack of guides for users, logistic and organizational barriers, and app-related and technical issues.

**Conclusions:**

Health professionals and patients found ADOC to be a valuable tool when setting shared rehabilitation goals. The use of ADOC promoted a patient-centered approach that empowered patients to engage in collaborative goal setting. The technological limitations of the app that negatively impacted experiences can be addressed in the future implementation of ADOC in rehabilitation settings.

## Introduction

### Background

Goal setting is a key part of the rehabilitation process [1] and is ultimately geared toward helping patients make functional progress in their recovery [2]. Rehabilitation goals have been defined as “a desired future state to be achieved by a person with a disability as a result of rehabilitation activities” [1]. Rehabilitation goals are “actively selected, intentionally created, have a purpose, and are shared-where possible-by the people participating in the activities and interventions designed to address the consequence of acquired disability” [1]. Goal setting has face validity as a method to enhance communication and collaboration within rehabilitation teams and may result in improved patient-reported quality of life after rehabilitation [1]. Research from psychology suggests that the right type of goals can have a significant effect on human performance across a wide range of activities [3]. It has been suggested that patient involvement in setting rehabilitation goals may lead to measurable improvements in physical and psychosocial function [2,4-6]. It has also been proposed that involving patients in decision-making may improve the quality and person-centeredness of rehabilitation practice. Collaborative decision-making aims to ensure that patients are well informed and meaningfully involved in choices about their care and that the treatments or interventions they receive reflect their goals and concerns [7,8].

**Figure 1:**
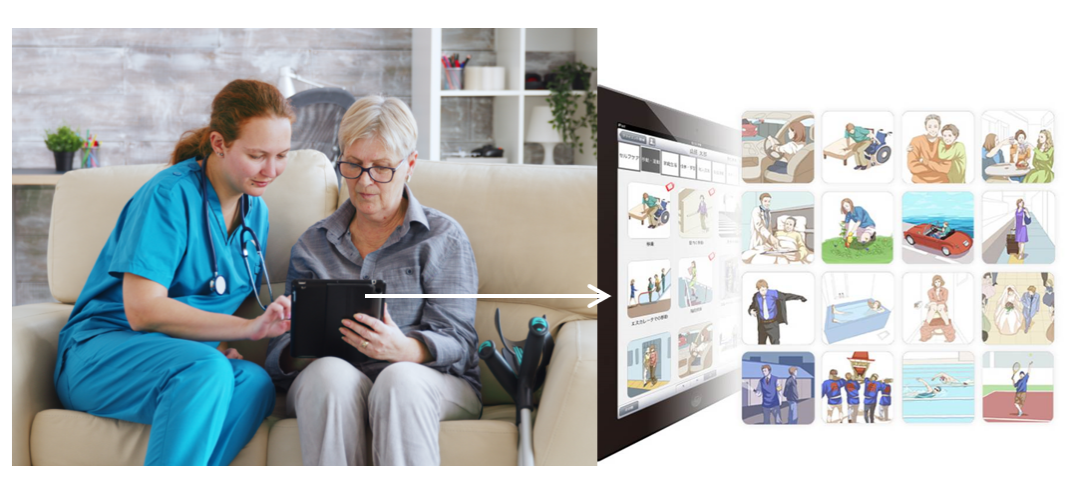
Example of a goal setting meeting using the iPad app Aid for Decision-making in Occupation Choice. Image source: Freepik [16]

**Figure 2:**
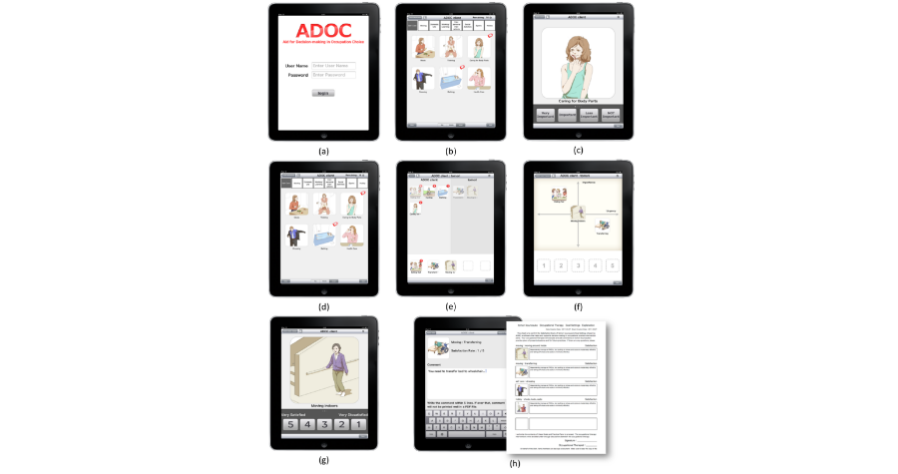
Main features of Aid for Decision-making in Occupation Choice. (a) Log-in page; (b) images from which the patient chooses up to 20 meaningful activities; (c) the patient rates each selected activity by importance; (d) the health care professional chooses up to 20 of the most important activities for the patient; (e) shared-decision moment, when the patient and the health care professional choose together up to 5 of the most urgent goals; (f) matrix page to prioritize the 5 goals by importance and urgency; (g) satisfaction rate page; (h) therapy plan in PDF format.

The use of technology and electronic tools such as smartphone apps and websites has been suggested as a way of improving the engagement of users in meaningful goal setting and facilitating shared decision-making between patients and health professionals [2,9,10]. The Aid for Decision-making in Occupation Choice (ADOC) [11] is an iPad app that was developed in Japan and designed for people with any disability; it helps patients identify and express the desired activities and social roles they want to work toward during rehabilitation, and it encourages them to participate in the goal-setting process [5]. ADOC uses texts and illustrations to present goal topics based on everyday activities and social roles, drawn from the *activities and participation* domain of the International Classification of Human Functioning, Disability, and Health [12] (Figures 1 and 2). The patient satisfaction scores derived from the Japanese version of ADOC are valid and reliable [13], and patients with moderate cognitive impairment can use ADOC to communicate their preferences for meaningful areas of activity [14].

In 2018, an English language version of ADOC was developed in consultation with 14 experienced international occupational therapists (OTs) [15]. This version of ADOC changed the language used, but also revised some illustrations and the range of goals to align with westernized activities and social roles. Early testing of this content showed that most of the images in the English language version of ADOC could be identified correctly by rehabilitation or residential care service users as a fair representation of the concept they intended to represent [15]. To date, ADOC has been tested extensively in clinical rehabilitation practice in Japan and has been demonstrated to support OTs in setting person-centered goals [5]. Both Japanese and English versions of ADOC have been tested and are validated for patients with any health condition, chronic or acute, and disability who score more than 9 on the Mini Mental State Examination scale [14]. However, although the Japanese version of ADOC was designed by OTs for OTs and has only ever been tested in this context, we were also interested in the potential for ADOC to be used for goal setting by staff in a multidisciplinary rehabilitation team.

### Prior Work

In 2020, we conducted a scoping review of the use of technology for goal setting in health care and found that ADOC was 1 of just 5 mobile apps or websites that supported collaborative decision-making between health professionals and patients for goal setting. Of these 5 apps, ADOC was the only technology that focused on the shared decision moment and that could be used in an interprofessional rehabilitation context for patients with any type of health condition [17]. We were therefore interested in the potential for ADOC to facilitate shared decision-making around goal setting in an English-speaking country and a wider group of health care personnel in rehabilitation. As this app had not been previously studied in this context, we chose a qualitative, open-ended approach to explore its potential use.

### Study Aim

The objective of this study is to investigate the experiences of health professionals and patients in the use of the English language version of ADOC to facilitate collaborative goal setting in English-speaking rehabilitation services. In particular, we wanted to understand what health professionals and patients liked and did not like about ADOC; how ADOC aligns with other clinical processes and practices; how ADOC can be incorporated into clinical practice; how ADOC influences clinical decision-making in an everyday rehabilitation setting; and what patient outcomes ADOC might most affect.

## Methods

### Study Design

We used a qualitative descriptive study design [18]. We collected and analyzed data, using semistructured interviews, on the perspectives of participants involved in trialing ADOC in an inpatient rehabilitation setting. This allowed us to not only collect data targeting our initial research questions but also enabled patients and health professionals the flexibility to elaborate on their views on the use of ADOC during the goal-setting process [19,20]. This study received ethical approval from the Northern B Health and Disability Ethics Committee, Ministry of Health, Wellington, New Zealand (reference number: 20NTB40) before participant recruitment. This paper presents the findings following the Consolidated Criteria for Reporting Qualitative Studies guidelines (see Multimedia Appendix 1). The research team included academic researchers with extensive experience in qualitative methods and technology: a physiotherapist (WMML), a rheumatologist (RG), 2 OTs (K Tomori and K Takashi), and a PhD student with a professional background in physiotherapy (CS).

### Setting

The study was undertaken in 3 inpatient rehabilitation services in the Wellington and Auckland regions of New Zealand; 2 government-funded services in public hospitals, and 1 private rehabilitation service funded mostly by the New Zealand national health insurance system for accidents (the Accident Compensation Corporation).

### Participant Selection and Recruitment

We recruited both health professionals and patients. Service team leaders and health professionals were approached by the research team (CS and WMML) a few months before the study, provided with the research protocol, and asked if they were interested in participating in the study. Service team leaders then provided names of health professionals who were interested in the study.

To be included in the study, the health professionals had to be qualified and registered allied health professionals (physiotherapists, OTs, and speech-language therapists) who were involved in goal setting with patients in their rehabilitation service as part of their usual role. We used purposeful sampling [21] to ensure that the participating health professionals had diverse professional backgrounds, years of work experience, and place of employment. Health professionals were not remunerated for their contribution to the study; however, their service departments were given copies of ADOC for use on their own devices after the study at no cost.

Patients were eligible to participate if they were over 18 years of age, current recipients of hospital rehabilitation services, able to provide informed consent, and able to have a basic conversation in English about their views and experiences with at least simple phrases and words to communicate their perspectives. Patients with mild cognitive impairment were eligible to participate in the study if they had a score ≥3 in the Montreal Cognitive Assessment [14,22] or a score ≥21 in the Mini—Addenbrooke Cognitive Examination [23-25]. Type of injury or illness and time, as injury or illness onset were not reasons for exclusion. All patients participating in the study were offered a New Zealand $20 (US $15) retail voucher as thanks for their participation. Patients were purposively sampled to include men and women, people from a range of age groups and ethnicities, and with different levels of cognitive ability.

### Materials and Training

Each rehabilitation service was provided with either an Apple iPad with ADOC already installed or the primary investigator installed ADOC on a service-owned iPad. ADOC is available only in the Apple store and only for iPads. Health professionals’ participants met with the primary investigator (CS) for in-person or web-based group training in the use of ADOC. The training was conducted in person in June 2020 for both the public hospital and the private rehabilitation center in Wellington. The in-person training was held in the rehabilitation service staff room, lasted approximately 2 hours, and primarily focused on how to navigate through the app and its functions. Owing to the geographic distance, training for Auckland Hospital was conducted on the web via videoconference in August 2020. The training was conducted in each location 3 to 4 weeks before data collection began. During the training, each health professional was able to try out the app and to ask questions. As we were interested to know how intuitive ADOC was to use and how health professionals might choose to use the app when this decision was left up to them, we kept instructions on when and how to use it to a minimum. We asked the health professionals to use ADOC with patients in their service as part of their usual goal-setting process in any way they saw fit.

### Data Collection

We collected data using individual semistructured, open-ended interviews with all participants between June 2020 and November 2020. Two interviews were conducted for each health professional and one interview for each patient. All interviews were scheduled and conducted by the primary investigator (CS). Interviews typically commenced with an open invitation for participants to describe their initial understanding of ADOC, what they like or did not like about the app, and their thoughts and feelings about using the app in clinical practice. Interview schedules with broad areas for questioning were used for all interviews (see Multimedia Appendices 2 and 3). The interviews could also develop organically, according to each participant’s responses. All interviews were audio-recorded using a high-quality digital recorder and transcribed verbatim.

The interviews with health professionals were performed in person at their place of work or on the web by videoconference. The first interview occurred within 7 days of the start of their use of ADOC and the second interview 4-6 weeks later. Each health professional provided information on their age, gender, professional role, and years of professional work experience.

Patients were interviewed in person or on the web by videoconference, within each rehabilitation service, in an appropriate, private, and comfortable room. The interviews were conducted within 10 days of using ADOC to set goals for their rehabilitation with their health professional. For each patient, we also gathered demographic and clinical information from the medical records including age, gender, ethnicity, current residential status, primary diagnosis, and Montreal Cognitive Assessment or mini-Addenbrooke Cognitive Examination scores. We continued recruiting participants and collecting data until we found that interviews were not identifying any new information, that is, when data saturation had been reached.

### Data Analysis

Data coding, following constant comparative methods, was used to explore and better understand the meaning of the information provided by participants [26-28]. We used NVivo software (QSR International) to manage data analysis. The transcribed interviews were systematically reviewed by 2 principal researchers independently (CS and WMML) who manually coded, identified, and categorized themes to familiarize themselves with the data and to enhance the richness and trustworthiness of the analysis process and findings. The other researchers also checked some sections of the transcripts for accuracy in coding. In cases of disagreement, codes were discussed until consensus was reached. An open coding process (fracturing of the data and grouping and categorizing) was used, so codes were not preset but developed and modified during the coding process [29]. The participants’ own words were used to guide the construction of codes and their definitions [30] and to enhance the credibility of the analysis. The analysis of health professionals and patient’s data were kept separate during the initial stages of analysis, but as the study progressed, we looked for commonalities and differences of ideas and experiences between the groups.

The trustworthiness of this study was ensured by enhancing its credibility, transferability, and dependability [31]. Credibility was achieved via research triangulation, using multiple analysts to review data sets, generate codes, and develop themes, to ensure that the research findings were robust, rich, and comprehensive. We addressed the transferability by providing a detailed description of the setting (private and public rehabilitation services in New Zealand) and the context (this study aims to analyze the experience of health professionals and patients in the use of an iPad app for goal setting in rehabilitation) in which this study took place. The reliability of this study was upheld by describing the research steps taken from the research protocol to the development and reporting of the findings. Anonymized extracts from the interviews are presented in the results to illustrate key findings.

## Results

### Overview

A total of 8 health professionals (see Table 1) and 8 patients (see Table 2) participated in this study. All participant interviews were conducted between June 2020 and November 2020 and lasted between 5 and 30 minutes (mean interview with patients 10.46 minutes, SD=5.22; mean first interview with health professionals 14:51 minutes, SD=5.23; mean second interview with health professionals 13:37 minutes, SD=7.08). All patients were inpatients in an acute rehabilitation ward, who had been hospitalized with a diagnosis of traumatic brain injury, stroke, chronic ulcer leg, or wound skin graft. None of the participants recruited dropped out from the study. Six main themes were identified from the analysis of the interview data. Overall, ADOC was seen as a valuable addition to the rehabilitation process by patients because it helped them broaden their understanding of what rehabilitation could potentially be about and what they could discuss with their health professionals as outcomes they wanted to work toward (theme a). Health professionals valued ADOC because it had the potential to change or enrich their understanding of what type of goals might be more meaningful or important to their patients (theme b). Thus, ADOC facilitated conversations around personally meaningful goals and person-centered goal setting (theme c). However, health professionals and patients also indicated that there were limitations to ADOC. These limitations were grouped into 3 main themes: problems with the lack of guides in the form of a user manual on how to use the app in clinical practice and printed material of the illustrations goals for patients (theme d), logistical and organizational problems that limited the use of ADOC in clinical practice (theme e), and problems with aspects of the design of the app or with its interface with the localities’ information technology systems (theme f). Each of these themes is discussed in more detail.

**Table 1:** Characteristics of health professionals interviewed (n=8).

Characteristics		Value, n (%)
**Gender**		
	Female	7 (87)
	Male	1 (13)
**Age (years)**		
	18-34	6 (75)
	≥35	2 (25)
**Role**		
	PT^a^	3 (37)
	OT^b^	3 (37)
	SLT^c^	2 (25)
**Work experience (years**)		
	<5	4 (50)
	5-10	2 (25)
	>10	2 (25)
**Work setting**		
	Wellington Public Hospital	3 (37)^d^
	Wellington Private Rehabilitation Service	2 (25)^e^
	Auckland Public Hospital	3 (37)^f^

^a^PT: physiotherapist.

^b^OT: occupational therapist.

^c^SLT: speech-language therapist.

^d^2 physical therapists and 1 occupational therapist.

^e^1 occupational therapist and 1 speech-language therapist.

^f^1 physical therapist, 1 occupational therapist, and 1 speech-language therapist.

**Table 2:** Characteristics of patients interviewed (n=8).

Characteristics		Value, n (%)
**Gender (n=8)**		
	Female	3 (37)
	Male	5 (63)
**Age (years, n=8)**		
	18-64	6 (75)
	≥65	2 (25)
**Ethnicity (ETHNIC05^a^, n=8)**		
	Māori	1 (13)
	New Zealand European	6 (75)
	Pacific peoples	1 (13)
**Primary diagnosis (n=8)**		
	Stroke	3 (37)
	Traumatic brain injury	3 (37)
	Wound skin graft	1 (13)
	Chronic ulcers leg	1 (13)
**Montreal Cognitive Assessment** **score (n=3)**		
	23/30	2 (67)
	26/30	1 (33)
**Mini-Addenbrooke Cognitive Examination score (n=5)**		
	27/30	2 (40)
	28/30	1 (20)
	29/30	1 (20)
	30/30	1 (20)
**Setting (n=8)**		
	Wellington Public Hospital	3 (37)
	Wellington Private Rehabilitation Service	3 (37)
	Auckland Public Hospital	2 (25)

^a^ETHNIC05: Ethnicity New Zealand Standard Classification 2005, V2.1.0.

### Theme a: Changing Patients’ Perspective on What Is Possible

All participating patients remembered using the app with their health professionals during the goal-setting meeting and for most of them, the initial experience with ADOC was regarded as positive. The app was described as “relatively easy to use*”* [P3]*,* “worthwhile” [P4], and “straightforward” [P5]. Because of the context of the research, where the patients were in an acute ward hospitalized with a severe condition, most of them did not know what to expect from the rehabilitation process. Accordingly, they did not know what goals were potentially possible to discuss during their hospitalization period or to achieve following it. The ADOC app helped patients to have a better understanding of the treatment expectancy and gave them hope for their potential recovery:

It really did help in having those choices put in front of me and not having to think about them, it made you realize that you know you could get there eventually.

P 8

### Theme b: Changing Health Professionals’ Perspective on What Is Important

This theme relates to the health professionals’ perception of what is meaningful to patients when setting rehabilitation goals. All health professionals had an overall positive first experience with using ADOC, which was described as “valuable” [HP8, first interview, “straightforward” [HP2, second interview], “easy to use” [HP4, first interview], and as “a good tool” [HP3, second interview] to support goal setting with patients. Goal setting was described as a complex conversation to have with patients, which ADOC helped them navigate:

[ADOC] it’s a nice way to approach a difficult discussion. What I really like about ADOC is that allows you to explore what they [clients] feel is important to them...because sometimes the stuff that the clients feel and the stuff that the therapists want to or perceive for the client are quite different.

HP5, first interview

In addition, most of the health professionals expressed, both during the first and second interviews, that ADOC had the potential to promote a more patient-centered approach to goal setting. They identified that the patient-centeredness model was essential and fundamental to a strong relationship with patients but was sometimes overlooked for various reasons, such as time. Health professionals stated that ADOC had the potential to reinforce engagement and provide prompts to the discussion around goal setting with their patient. Health professionals strongly expressed the view that ADOC reinforced their patient-centered approach in clinical practice while setting rehabilitation goals:

It was really good just learning more about the client and just asking them different goals. I think usually I focus on what I think they kind of need to do to get home.

HP3, second interview

If we can find out from their viewpoint what their goals are that may help them actually feel some ownership.

HP, second interview

I feel like it [ADOC] definitely improves the whole client-centered approach.

HP8, second interview

Almost all health professionals were positive about using ADOC in their clinical practice in the future; however, all agreed that they would not use ADOC with every patient. Health professionals stated that ADOC was not appropriate to use with patients with severe cognitive impairment or with patients who were already clear about and able to easily express their goals for rehabilitation:

I think it has to be a certain type of client though…like it honestly doesn’t work with everyone.

HP7, first interview

I have recently had a lot of clients with cognitive impairment and a lot of them would not have been appropriate.

HP7, second interview

So, I think it’s good for people who just have no idea what sort of goals to set so they can sort of look through and brainstorm what’s important to them.

HP8, second interview

Finally, few health professionals expressed the view that they would have set the same goals with or without ADOC. They suggested that ADOC was a good device to initiate a “difficult discussion” [HP5, first interview] and to help them “identify the importance of which goals the client wanted to work on” [HP4, first interview] but that otherwise ADOC would not support identifying unique or different goals.

I don’t think that the end result changes.

HP2, first interview

I don’t feel that I necessarily got any extra goals that wouldn’t have come out from the standard goalsetting process.

HP8, second interview

### Theme c: Facilitating Shared Decision-Making

Overall, most of the health professionals thought that ADOC facilitated their decision-making process and the identification of meaningful goals for their patients. Some health professionals reported that goals that were important to patients were sometimes overlooked during their usual goal-setting practice without ADOC. They also said that ADOC was helpful because it allowed identifying the most significant goals for the patient in a shared environment, which facilitated a shared purpose and prioritization:

For me, I missed that goal [toileting], but it was identified with ADOC.

HP5, first interview

It was really good because we would never have thought of that [goal], well I would have never thought about it really before.

HP7, first interview

He picked sleeping to be his number one priority which was interesting because obviously that’s not necessarily something I think of.

HP8, first interview

Most patients reported that ADOC improved the communication with their health professional, facilitated by the accompanying images. Having the option to decide which goal to work toward from a predetermined list made patients feel more empowered and more confident. Mostly the visual aspect of ADOC, where all the goals are illustrated by a deliberately designed image, was a key advantage for the patient. The images prompted and generated conversation, favored the patients’ perspective when communicating with their health professionals, and motivated patients to strive for success in their rehabilitation. ADOC was defined as a very good tool for those patients that “want to get better but don’t realize the potential they have” [P8]:

You know, not just for me but for a lot of the clients in here, images tell a thousand words.

P1

The health professionals also valued the wide range of images used to represent the goals, which were seen as “helpful” [HP8, first interview] and as “support for their [patients] comprehension”

HP6, second interview

Especially for my clients having that visual prompt or sort of like support for them gives them a better understanding of what they’re discussing when it comes to goal.

HP6, second interview

### Theme d: Lack of Guides for Users

The health professionals also identified several areas where improvements could be made to ADOC and its application to goal setting. They commented on the lack of technical guides or documentation to support the use of ADOC. Although training was provided by the primary investigator at the beginning of this study, the health professionals expressed that it would have been useful to have a user manual or prompt sheet containing all essential information and step-by-step procedures for app access and use. Some health professionals stated that although ADOC was quite intuitive, a user manual would still have been convenient so that it could be consulted whenever doubts arose:

It would be good to have a prompt sheet for the therapist to use with like a script to avoid any confusion when you’re explaining it [ADOC] to the client.

HP7, first interview

The second problem described was the absence of a visual guide that showed all the goal illustrations for patient users in a hard copy. Some patients stated that they would have preferred to look at the images of goals using a hard copy format before using the app, to increase their confidence in app use, to have as much time as needed to analyze the most meaningful goals, and to understand the total time required to scan each goal. Some patients felt “overwhelmed” [P1] and “frustrated” [P4] by the extent of content in ADOC and found the app “too long” [P4]. These patients also highlighted their lack of confidence in using technology in general. The health professionals also agreed some patients would benefit from reviewing all the goal images in hard copy before using the ADOC app:

I didn’t really know the size of it [ADOC] because it wasn’t in hard copy so I didn’t really know what was coming, if there was a [hard] copy I would be able to just flick through and go okay I can get an idea of what this is about.

P4

A hard copy might be quite nice that they [patients] could look through first and then when you came to do the goal-setting process, they were more familiar with all the symbols and everything.

HP8, second interview

### Theme e: Logistic and Organizational Barriers

This theme relates to all organizational and logistical issues that limited the use of ADOC in clinical practice. For instance, the health professional identified that while they had been invited to use ADOC with as many patients as possible, use of ADOC was limited by simple matters such as knowing where the organization’s iPads were stored and being able to access them easily when they wanted one:

It’s just actually the accessibility of the iPad and where it is and so if it’s like in your visual field day-to-day, you’re more likely to use it. So, I think having one iPad that’s shared between both wards with multiple people on it is a little bit of a barrier with it.

HP2, second interview

However, the key reason limiting the use of ADOC was the degree to which health professionals could prioritize the time required to use the app effectively set rehabilitation goals in practice. ADOC was considered by most of the health professionals as “time consuming” [HP4, second interview] and “not feasible” [HP5, second interview) to use regularly in a hectic work environment:

It took a long time with that client. It took a whole 60-minute session. It takes longer than I anticipate it will take.

HP7, second interview

It just adds time just been really stressed and I’ve been really stressed for time this last couple of weeks.

HP8, second interview

Therefore, the health professionals suggested that ADOC might have better utility in a community-based rehabilitation service, where patients receive rehabilitation over a longer period than in an acute setting and where, they believed, health professionals have more time to spend with their patients during goal-setting meetings:

It definitely works [better] closer to discharge, and it would work really nicely in the community.

HP2, second interview

Moreover, some health professionals stated that the number of long-term goals illustrated in the app was higher than the number of short-term goals and that therefore community-based rehabilitation services would probably benefit more from the app:

A lot of the goals are really nice but they’re very much community more goals, like longer-term.

HP7, second interview

### Theme f: App-Related Problems and Technical Issues

The health professionals noted that some goals they wanted to set were not available in the app, such as goals related to “mental health” [HP5, first interview], “memory” [HP4, first interview], and “managing pain” [HP4, second interview]. Of note, ADOC was specifically designed to focus goal setting toward functioning at the level of activities and participation and intentionally omits goals at the level of impairments of body structure and function; however, some health professionals nevertheless wanted to set impairment-oriented goals. Patients also noted these and other types of goals as being absent and included the ability to “multitask” [P8], or “manage grief and depression” [P3]. Both health professionals and patients suggested ADOC be improved by the option to add personalized goals, especially useful for those people who have “unusual jobs or hobbies” [P8]:

We [health professionals] just wondered whether there were some options, which might be really useful for people especially people who have traumatic brain injuries around managing frustration or managing behavior. The other ones that come up for us a lot is memory and concentration, those are quite big goals for a lot of people after they’ve had a brain injury. And we also talked about one having an option for something around kind of dealing with grief or something around feelings.

HP4, first interview

Moreover, some health professionals highlighted that the images in the app (which had been drawn in Japan) were not representative of the multicultural make-up of New Zealand. There was a desire among the health professionals to have images to show patients to more accurately reflected the ethnicities of the people they worked with:

The images aren’t multicultural. They are all sort of Asian based pictures which is fine, but you may have some people that would like to see their ethnicity represented.

HP2, first interview

I think the pictures are helpful, but I think when you get ones that are maybe more culturally appropriate for New Zealand, I think that that would be really helpful.

HP8, first interview

Furthermore, the health professionals identified a few technical issues, which seemed to have hindered the use of ADOC in everyday practice. These technical issues included the lack of an interface between the app and their organization’s hardware and systems. Examples included not being able to access the PDF treatment plan and not being able to email it to their work email or print it from their organizational printer:

I think one of the things that we had difficulty with is getting access to just printing the list of goals off. It’s just a bit trickier process when it’s the company’s device we have to go through IT to organize it.

PH4, second interview

## Discussion

### Principal Findings

This study found that overall ADOC was accepted and liked by both health professionals and patients as a tool for supporting shared decision-making for goal setting in rehabilitation, although some barriers to its implementation in clinical practice were identified. The aspects of the app that were most valued were its practical utility, that it promoted a patient-centered approach to goal setting, and that it facilitated communication between health professionals and participants about the objectives and direction of rehabilitation. This is the first study to show the utility and potential value of ADOC when used in an interprofessional context rather than solely in an occupational therapy context. These findings suggest that ADOC has the potential to be incorporated into clinical practice and be used by multidisciplinary teams. In this study, ADOC was valued by most of the patient participants because it enabled them to have a better understanding of what to expect from rehabilitation and therefore it empowered them to be more involved in meaningful decisions about their care. This aligns with the known benefits of patient participation in health care decision-making, which include increased patient satisfaction and trust, a better understanding of personal requirements, more positive communication with health professionals, increased sense of self-responsibility, and has implications for ongoing motivation, autonomy, and adherence to behaviors [32-34].

Our findings also emphasized the importance of a patient-centered approach in rehabilitation. Health professionals stated that ADOC promoted a more patient-centered approach when compared with their usual goal-setting practice; the app highlighted the value of building a better understanding of their patients’ preferences and priorities. As patient-centeredness seems to be positively associated with higher levels of patient satisfaction and may improve treatment outcomes, health, and psychological well-being [35], this is a desirable benefit as a result of using ADOC. The health professionals in this study also identified several shortcomings of ADOC or challenges in its application to clinical practice. These included the increased time needed to engage in goal setting, the lack of representativeness of illustrations to reflect a New Zealand population, and the lack of a written guide for users, which was perceived to be necessary.

We are currently working on a version of ADOC that includes images and content that reflects a more ethnically diverse population, with specific attention to the representation of Māori and Pacific people who collectively make up almost 25% of the New Zealand population [36]. We have also developed more detailed guidebooks on the use of ADOC in clinical practice, which will be tested in future studies. Issues around the time taken to undertake goal setting are more challenging to address as this relates to prioritizations of activities to support person-centeredness in the clinical setting. It is widely acknowledged that the adoption of new technologies can be hindered by insufficient training and education support for health care professionals [37,38]. Zheng et al [39], argued that health care professionals may find mobile health technologies disruptive to workflow when they do not complement work habits, when they create additional work, or when they present changes to familiar routines. The participants in this study reported that having easy and immediate access to iPad devices in their workplace and more time to dedicate to the goal-setting session with the patient would have facilitated the use of ADOC. They also speculated that ADOC may be more suited to use in community rehabilitation settings.

To date, there has been limited research comparing the use of technology in acute rehabilitation settings versus community rehabilitation settings. Therefore, future research regarding technology to support goal setting in a community-based rehabilitation setting is needed. Future implementation of such software should proactively address the barriers to the update of new technology identified in this study, particularly the need to integrate new technology with existing organizational processes. Finally, some of the health professionals in this study viewed the change of goal setting from an interview process to an interactive process as unhelpful. It has previously been recognized that individuals unwilling to change behavior practices and adopt new solutions into their workflow can obstruct the uptake of innovative technologies [40]. Therefore, identification of these people and strategies to address their concerns are needed if new technology is to be successfully implemented in practice.

### Strengths and Limitations

A strength of this study is that it included a variety of health care professionals who specialize in rehabilitative care in testing ADOC in clinical practice. Previously, ADOC has only been tested and used by OTs in Japan. The qualitative approach also allowed a detailed exploration of users’ experiences of ADOC in rehabilitation settings, producing information that can guide future research and implementation of this technology in clinical rehabilitation. Conversely, this study only involved a small number of health professionals and patients, so the transferability of these findings still needs testing. We also did not design this study to explore whether there was any clinical benefit for use of ADOC for goal setting in rehabilitation. A clinical trial design would be necessary to draw provisional conclusions about the comparative treatment effect of a different approach to goal setting.

We also did not ask health professionals about their familiarity with technology before the study or their general views on technology adoption. As the interviews with health professionals did not highlight any views about difficulties in engaging with ADOC, we assumed that the health professional participants in this study were those with a generally positive attitude toward the use of technology in their practice. Therefore, we acknowledge that selection bias may have influenced our findings, which should be interpreted with this caveat. Future research should aim to recruit health professionals less keen and skilled in the use of technology in clinical practice. We also reported that access to iPads was a concern for health professionals, limiting the use of the app in goal setting. We ensured each locality was loaned two iPads if none were available onsite or we installed ADOC onto service-owned iPads, assuming that a few iPads could be easily shared within an interprofessional team. However, it would be desirable in future research to ensure that all health professionals always have access to an iPad each when working clinically if testing the utility or benefits of ADOC. It has been widely stated that research should focus on producing and developing innovative technologies for integration into the health care system [5,15,41,42]. Our study suggests incorporating technology use into clinical practice remains challenging and attention to nontechnology-related barriers will be necessary to maximize the potential for digital health technology to improve quality of service delivery, patient satisfaction, and health outcomes.

### Conclusions

On the basis of the results of this study, the iPad app ADOC has been shown to be a valuable tool for health professionals and patients while setting shared rehabilitation goals. As the study was exploratory and conducted with a small sample size, we believe that future research is needed to further understand the potential for ADOC to be a suitable app for supporting goal setting in the context of interdisciplinary rehabilitation. It is also crucial that future research further explores organizational, logistic, and technical barriers and addresses these to improve the potential benefit of ADOC.

## Appendix

Multimedia Appendix 1 Consolidated Criteria for Reporting Qualitative Studies.

Multimedia Appendix 2 Interview schedule with health professionals.

Multimedia Appendix 3 Interview schedule with patients.

## Abbreviations

ADOC: Aid for Decision-making in Occupational Choice
OT: occupational therapist
