# Supplementary material for: Use of an iPad App (Aid for Decision-making in Occupational Choice) for Collaborative Goal Setting in Interprofessional Rehabilitation: Qualitative Descriptive Study
Source: JMIR Rehabil Assist Technol. 2021 Nov 18;8(4):e33027. doi: 10.2196/33027 (PMC8663657; doi:10.2196/33027)
Supplement: Multimedia Appendix 2 [file rehab_v8i4e33027_app2.docx]

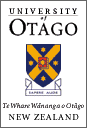


**INTERVIEW SCHEDULE WITH HEALTH PROFESSIONALS – 1^st^ interview**

Kia ora,

My name is Carla Strubbia and I am a PhD student at the Department of Medicine at the University of Otago, Wellington. I am also the principal investigator of this study. During this interview I would like to ask you few questions about your view on ADOC, how useful you found it and what you liked or didn’t like about it. I will record you, as it was specified in the consent form that you signed, so I can make sure I do not miss anything important that you say. I will use this information and any other comments you make to understand whether ADOC should be part of the clinical practice or not. The interview should take about 10 to 30 minutes, but we can stop or have a break at any time as you need.

- What ADOC was like to use?
  - - What did you like?
    - What did you not like?
    - What do you think might make it more useful?
- Did ADOC influence your usual practice?
  - - How did ADOC align with the other clinical processes and practices?
    - How can ADOC be usefully incorporate into your clinical practice?
    - Is there anything you would have done differently in your usual clinical practice without ADOC?
- Did ADOC influence your clinical decision-making?
  - - If yes, how? If not, why?
- Did ADOC influence your goal setting meeting?
  - - What influence did ADOC have in the types of goals that were identified?
    - Do you think you chose different goals than you would normally do?
    - Did you check all categories and goals one by one or did you focus more on specific ones? If yes, why?
    - Did you find the patient was more engaged than usual during goal setting meeting?
- Did ADOC change or effect the interaction with your patient?
  - - If yes or not, please explain why?
    - Do you think ADOC helped the communication between you and your patient?
- What do you think is the added benefit/value of ADOC?
- Why would you choose ADOC instead of normal practice?
- Is there anything you would like to add before I complete this interview?

The interview is finished.

Thank you very much for your time, patience and for your precious collaboration in this project. Please feel free to contact me at any time if you have any question now or in the future.

Ngā mihi,

Carla Strubbia

**INTERVIEW SCHEDULE WITH HEALTH PROFESSIONALS – 2^nd^ interview**

- Have you had any opportunity to use ADOC again in the last month?
  - - If yes, how has been gone?
    - If not, Why?
- Would you like to tell me again what you did you like and what you did not like about ADOC?
- What impact did ADOC have in your usual clinical practice?
- Would you like to incorporate ADOC in your clinical practice in the future?
  - - If yes/not, why?
- Is there anything that came out of the goal setting discussion using ADOC that otherwise would have not came out?
- Do you think ADOC had a role in your decision making about which goal to select?
  - - If yes/no, why?
- Could you please tell me how do you usually do goal setting in normal practice and what was the main difference for you when using ADOC?

The interview is finished.

Thank you very much for your time, patience and for your precious collaboration in this project. Please feel free to contact me at any time if you have any question now or in the future.

Ngā mihi, Carla Strubbia
