# Supplementary material for: Use of an iPad App (Aid for Decision-making in Occupational Choice) for Collaborative Goal Setting in Interprofessional Rehabilitation: Qualitative Descriptive Study
Source: JMIR Rehabil Assist Technol. 2021 Nov 18;8(4):e33027. doi: 10.2196/33027 (PMC8663657; doi:10.2196/33027)
Supplement: Multimedia Appendix 3 [file rehab_v8i4e33027_app3.docx]

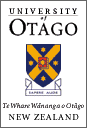


**INTERVIEW SCHEDULE WITH PATIENTS**

Kia ora,

My name is Carla Strubbia and I am a PhD student at the Department of Medicine at the University of Otago, Wellington. I am also the principal investigator of this study. During this interview I would like to ask you few questions about your view on ADOC, how useful you found it and what you liked or didn’t like about it. I will record you, as it was specified in the consent form that you signed, so I can make sure I do not miss anything important that you say. I will use this information and any other comments you make to understand whether ADOC should be part of the clinical practice or not. The interview should take about 10 to 30 minutes, but we can stop or have a break at any time as you need.

- Do you remember the therapist showing you this app called ADOC?

If NO:

- Tell me what you remember about your experience doing rehabilitation?

If YES:

- How was your overall experience of using this app?
  - - How did you feel?
    - Why do you think you felt that way?
- What did you like about using ADOC?
  - - What did you like, find useful or enjoyable about this app?
    - What did you not like about the app?
    - Could you please tell me why?
- Tell me more about your therapy goals and expectations?
  - - Do you remember setting goals?
    - How was the communication between you and your therapist?
    - Do you think you would have told the same goals to your therapist without the app?
    - Would you like to use this app again in the future?
    - Do you have anything else to add to this interview?

The interview is finished.

Thank you very much for your time, patience and for your collaboration in this project. Please feel free to contact me at any time if you have any question now or in the future.

Ngā mihi, Carla Strubbia
